# Tissue Engineering Therapies Based on Folic Acid and Other Vitamin B Derivatives. Functional Mechanisms and Current Applications in Regenerative Medicine

**DOI:** 10.3390/ijms19124068

**Published:** 2018-12-16

**Authors:** Daniel Fernández-Villa, Mirta Jiménez Gómez-Lavín, Cristina Abradelo, Julio San Román, Luis Rojo

**Affiliations:** 1 Instituto de Ciencia y Tecnología de Polímeros, Consejo Superior de Investigaciones Científicas, CSIC, 28006 Madrid, Spain; dafer-vi@hotmail.com (D.F.-V.); jsroman@ictp.csic.es (J.S.R.); 2 Consorcio Centro de Investigación Biomédica en Red de Bioingeniería, Biomateriales y Nanomedicina, 28029 Madrid, Spain; 3 Departamento de Química y Bioquímica. Facultad de Farmacia Universidad CEU San Pablo, 28668 Madrid, Spain; m.jimenez52@usp.ceu.es (M.J.G.-L.); abradelo@ceu.es (C.A.)

**Keywords:** folic acid, B-vitamin, tissue engineering, regenerative medicine

## Abstract

**** B-vitamins are a group of soluble vitamins which are cofactors of some of the enzymes involved in the metabolic pathways of carbohydrates, fats and proteins. These compounds participate in a number of functions as cardiovascular, brain or nervous systems. Folic acid is described as an accessible and multifunctional niche component that can be used safely, even combined with other compounds, which gives it high versatility. Also, due to its non-toxicity and great stability, folic acid has attracted much attention from researchers in the biomedical and bioengineering area, with an increasing number of works directed at using folic acid and its derivatives in tissue engineering therapies as well as regenerative medicine. Thus, this review provides an updated discussion about the most relevant advances achieved during the last five years, where folic acid and other vitamins B have been used as key bioactive compounds for enhancing the effectiveness of biomaterials’ performance and biological functions for the regeneration of tissues and organs.

## 1. Introduction

Regenerative medicine is currently one of the most promising advances in medical treatment, aiming to replace or regenerate damaged tissues or organs in order to restore their normal functions [1]. Nowadays, many approaches have been proposed for achieving this goal, such as cell therapies or the implantation of cell-seeded scaffolds, which combines the principles of cell therapy and the use of biomaterials [2]. However, there are still many diseases without an effective medical solution, which are being researched using different approaches.

In this regard, naturally occurring vitamins are a group of organic compounds that are essential for the normal physiological functioning, but which cannot be synthesized by the human body and thus, depend on a variety of dietary sources [3]. Specifically, B-vitamins are a group of soluble vitamins that are cofactors of some of the enzymes involved in the metabolic pathways of carbohydrates, fats and proteins, and due to their promising properties, many researchers have studied the advantages of their use in the clinical field, such as boosting the immune system [4], treating and preventing Alzheimer’s disease [5], the effects on brain function [3] or in bone health [6]. Others are currently developing different novel biomaterials based on B-vitamins such as polyphosphazenes [7], star polymers [8], nanoparticles [9] and nanocomposites [10] among others, in order to improve their biocompatibility or to add the effects of B-vitamins to the regeneration process.

The B-vitamin group includes thiamine (vitamin B_1_), riboflavin (vitamin B_2_), niacin (vitamin B_3_), pantothenic acid (vitamin B_5_), folic acid (vitamin B_9_) and cobalamin (vitamin B_12_), among others and each has a specific role in each tissue [6]. In particular, interest in the health benefits of folic acid has increased considerably over the past decade. This is in part due to its classical role in important biosynthetic pathways such as purine, pyrimidine and some amino acids syntheses and in maintaining the normal cell growth and replication processes [11], but also because of its more recently described epigenetic role [12,13,14].

Therefore, the main objective of this review is to update the state-of-the-art B-vitamin-based regenerative therapies and tissue engineering processes, as it is believed they have promising properties in several tissues, and to focus in detail on folic acid as the principal component of this group of molecules.

## 2. Results

**Figure 1:**
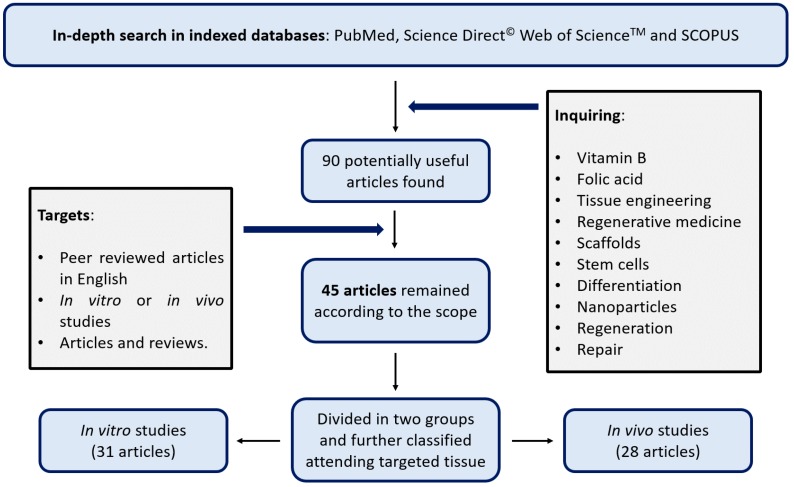
Flow diagram of the screening process carried out on this review.

As shown in Figure 1, after applying the search criteria, 45 articles were included in the review. These papers were classified according to whether the experiments were carried out in “in vitro studies” (31 articles) and “in vivo studies” (28 articles). Fourteen of these papers described both in vitro and in vivo studies. In vitro studies were mostly performed on human and murine cell lines and were focused on testing biomaterials’ cytotoxicity, biocompatibility, drug release, and biological properties of the cells such as changes in their proliferation rates, their differentiation commitment degree or their nanoparticle uptake, among others. In contrast, in vivo studies were more strictly focused on tissue regeneration, and quantifying the velocity and quality of these regenerative processes. In all cases, murine models were employed.

Additionally, in order to better organize the data, all the studies were classified in tables (Table 1 and Table 2) according to the target tissues: bone tissue (10 articles), inflammatory diseases (12 articles), the nervous system (15 articles), wound healing (5 articles), pancreas (1 article), and the cardiovascular system (2 articles).

## 3. Discussion: B-Vitamins as a Tool in Tissue Engineering

B-vitamins are good candidates for different tissue regeneration processes due to their proven biological role in regulating specific cell metabolism reactions and cellular communication processes. Many researchers are currently developing novel biomaterials by modifying existing ones using B-vitamins [57], and evaluating their bioactivity and mechanical properties in several tissues while exploring various biomedical applications. Since they can be applied in a wide range of tissues and targets, in this review, we discuss the different possibilities that these vitamins, particularly folic acid, offer in the regenerative and tissue engineering fields.

### 3.1. B-Vitamins in Bone Repair

**Figure 2:**
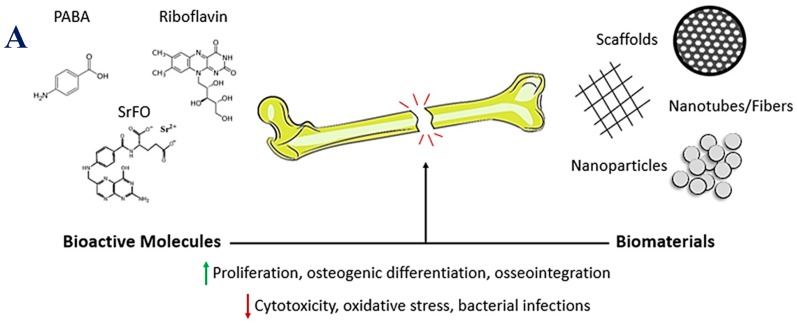

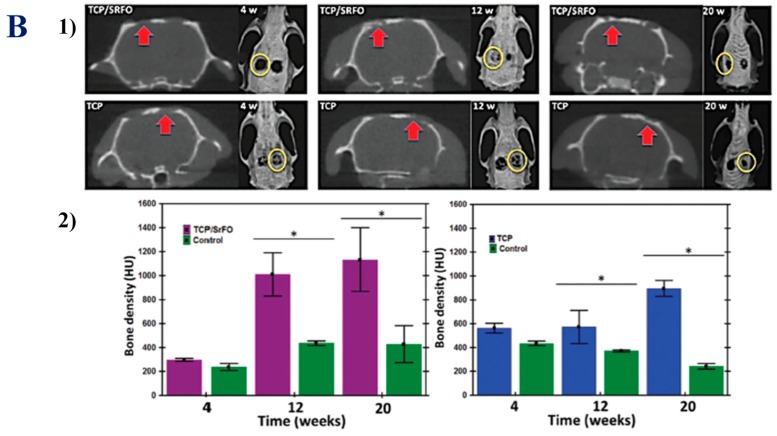
B-vitamins in bone repair. (**A**) Schematic diagram showing the different options to promote bone regeneration processes. Up-arrows mean enhanced. Down-arrows mean decreased. (**B**) Micro-computed tomography images of cranial defects treated with TCP and TCP/SrFO scaffolds at 4, 12 and 20 weeks and defect closure on the side of the implants form the coronal plane (arrows) and 3D images (circles) (**1**) and bone density of the radiographic density (HU) in cranial defects (**2**) (* *p* < 0.001). Figure 2B is reproduced from [16] with permission from the Royal Society of Chemistry.

Bone is a dynamic tissue which is constantly undergoing a remodeling process. The balance between bone synthesis and resorption must be finely regulated in order to ensure its mechanical integrity. Alterations in these physiological processes lead to bone augmented fragility, and consequently, to bone injury. Thus, when trying to regenerate bone, mechanisms for promoting accelerated osteogenesis or delayed bone resorption are sought (Figure 2A) [58].

Currently, strontium ranelate is used as a bone promoter for treating osteoporosis, particularly when patients do not tolerate other anti-osteoporotic drugs. However, due to the undesired side effects of ranelate moiety, Rojo et al. synthesized two folate compound derivatives of calcium (CaFO) and strontium (SrFO) [17]. These salts increased human osteoblasts (HOBs) cell viability within the range of 0.063–0.5 mg·mL^−1^ when compared with non-treated or folate-treated cells. These functionally active low doses are of particular interest because they fall within the physiologically active range of strontium (0.009–0.09 mg·mL^−1^). Therefore, SrFO exerted its effects by promoting higher and faster alkaline phosphatase (ALP) activity in treated groups. Furthermore, the formation of these complexes avoided the toxic effects of the free ions, as well as enhanced cell metabolism and replication processes.

Taking into account all those results, Martin-Del-Campo et al. developed a biohybrid scaffold of tricalcium phosphate (TCP), which was loaded with SrFO and seeded with human dental pulp stem cells [16]. They demonstrated that it enhanced the osteogenic differentiation of these cells and promoted bone formation in a rat calvarial defect model (Figure 2B). Although both TCP and TCP/SrFO groups exhibited progressive filling of the defect with mineralized tissue at 12 and 20 weeks after implantation, TCP/SrFO scaffolds had been completely resorbed, and newly formed bone had a mature appearance with a marked organized laminar trend and high bone density, while parts of some control TCP scaffolds group remained and there was non-lamellar bone in some areas.

Similarly, titanium (Ti) and its alloys have been used in a wide range of clinical applications such as orthopaedic and dental implants. Ti is known for being a stable (chemical and mechanically), non-toxic, bone integrating material. However, it does not fulfil the rapid osteointegrative demands needed for its clinical use [59,60]. Modifying the surface of Ti implants with bioactive molecules such as B-vitamins has been shown to improve the biocompatibility of bone implants. In this context, Lee et al. chose over 21 molecules to use pyridoxal 5′-phosphate (PLP), an active form of vitamin B_6_, to produce PLP-functionalized TiO_2_ [24]. They found that when in contact with human blood, PLP established links with platelets and plasma proteins such as albumin. On the one hand, PLP-platelets’ interaction induced a delay in blood coagulation and the release of growth factors from platelets such as platelet-derived growth factor, which is known to be involved in regulating MSCs differentiation towards osteoblastic lineage [61]. On the other hand, PLP-proteins’ interaction increased the availability of proteins on the surface of the implants, improving cell adhesion and proliferation. Taken all together, both approaches synergistically promote osteointegration and tissue repair.

On the other hand, Xu et al. functionalized titanium surfaces with SrFO to promote the osteogenic differentiation of mesenchymal stem cells (MSCs) and bone formation [15]. Folic acid was covalently attached on Ti substrates previously treated with hydrochloride acid and 3-(triethoxysilyl) propyl succinic anhydride, and SrCl_2_ was added to the solution to form the biomaterials. In vitro results showed that immobilized SrFO affected cell viability and improved cell proliferation in the long-term, it also enhanced the ALP activity of MSCs in the short-term and sustained upregulation for a long time. In addition, SrFO-implants on Sprague-Dawley rats femurs corrected the bone defect in eight weeks. There were no significant differences at four weeks between implants with Sr^2+^ on the surface and the SrFO-immobilized ones. The authors hypothesized that this could be due to the diffusion of Sr^2+^ when it is not immobilized, whereas the SrFO-implants had a sustained effect.

In every regeneration process, the local availability of folic acid is anticipated to improve cell response. For this reason, Santos et al. developed hydroxyapatite nanoparticles (HA NPs) which were loaded with folic acid (HA-FA NPs) to induce osteoblastic differentiation [18]. It was demonstrated that these nanoparticles were interiorized by MSCs by an endocytosis mechanism, not affecting their proliferation and viability but restricting their differentiation potential exclusively towards an osteogenic lineage, meaning that when a differentiation agent induces the differentiation process MSCs will only differentiate into osteoblasts. Both HA and HA-FA NPs were shown to increase the levels of ALP activity in a similar way, but HA-FA NPs also overexpressed the levels of *Runx2*, indicating a higher degree of commitment towards the osteogenic lineage. This approach can be applied to other B-vitamin examples such as riboflavin or its photoderivatives, which have been demonstrated to potentiate the differentiation of preosteoblastic line cell MC3T3-E1 towards a more mature osteoblastic phenotype [21], acting as adjuvants of ascorbic acid and β-glycerophosphate.

Shi et al. demonstrated the advantages of doping micro-grooved carbon fibers (CFs) with para-amino benzoic acid (PABA), a folic acid related metabolite, improving the strength and biocompatibility of polylactic acid-polyethylene glycol (PLA-PEG) [20]. The resulting biocomposite improved hydrophilicity while maintaining the basic tensile strength of the PLA-PEG surface. In particular, biocomposites with 0.5% CFs showed better results with no negative effects on the proliferation of two different line cells and with a good biocompatibility.

Other aspects to take into consideration when an implant is inserted to treat a bone defect include the possibility of bacterial infections as well as cytotoxicity. Xiang et al. developed a system to prevent Ti implants-associated bacterial infections [19]. Implants were composed of titania nanotubes (TNTs), loaded with an antibiotic (vancomycin), and capped by FA-QDs (ZnO quantum dots, which have been conjugated with folic acid on their surface). The system was stable under physiological conditions while the antibiotic was released, when the pH went down due to the bacterial metabolism. In addition, ZnO was degraded at those conditions into Zn^2+^, exerting an additional antibacterial effect.

On the other hand, one of the most common problems in tissue engineering is that excessive cell stimulation can cause cytotoxicity due to the generation of reactive oxygen species (ROS) such as hydrogen peroxide (H_2_O_2_), and consequently, lead to cell dysfunction and apoptosis. Thus, Ito et al. examined the effects of nicotinamide (NAM) in a cytotoxic-induced situation in rat bone marrow cells [22]. They used a H_2_O_2_ solution as the cytotoxic reagent and compared cells with and without a pre-treatment of NAM. When H_2_O_2_ was added, cells immediately displayed morphological changes (shrinking) and detachment. However, when cells were pre-treated with NAM, those changes did not occur and toxicity was delayed, suggesting that NAM regulates the timings of oxidative-stress-related cytotoxicity.

Likewise, Llorens et al. carried out an investigation on the benefits of adding antioxidants to biomaterials. They studied the effect of pyridoxine hydrochloride, pyridoxal hydrochloride, caffeic acid (3,4-dihydroxycinnamic acid) and *p*-coumaric acid (*p*-hydroxycinnamic acid) as antioxidants on polylactide (PLA) nanofibers [23]. Cell adhesion and viability of three different mammalian cell lines did not vary significantly in the presence of the scaffolds, indicating that antioxidants-loaded PLA matrices did not cause cytotoxicity. Altogether, these studies allowed us to conclude that B vitamins are emerging as good candidates to improve bone defect regeneration, as well as some of its complications.

### 3.2. B-Vitamins in Inflammatory Diseases

**Figure 3:**
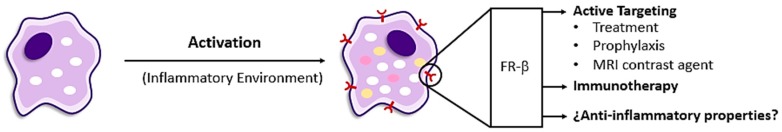
B-vitamins in inflammatory diseases. A schematic diagram summarizing the different mechanisms of action of folic acid for treatment or prophylaxis of inflammatory diseases.

Folic acid has been widely used in the oncology field as an active targeting agent for drug delivery due to folate receptor overexpression at the surface of cancer cells. However, it is well-known that activated macrophages also overexpress their folate receptor beta isoform (FR-β) in a pro-inflammatory environment [62]. Thus, an analogous active targeting mechanism could be used for treating diseases with an inflammatory component or autoimmune diseases (Figure 3).

Different vehicles have been designed for delivering drugs to inflamed regions. Poh et al. developed a folate-targeted dendrimer that selectively accumulated at inflammation zones (3 to 4-folds higher) in murine models of ulcerative colitis and atherosclerosis [29]. Dendrimers were made of polyamidoamine (PAMAM) and PEG and some were synthesized to carry fluorescent molecules in order to track their location in vivo. None of them exhibited cytotoxicity on RAW 264.7 cell line and they were demonstrated to deliver more cargo than small molecules.

Another drug delivery system which offers many opportunities are nanoparticles. In 2015, Cao et al. synthesized dexamethasone phosphate-loaded poly(lactic-co-glycolic acid) (PLGA) nanoparticles which were conjugated with folate in order to actively target activated macrophages [30]. It was the first report of glucocorticoid selective delivery against this cellular population and the first study that addressed the effect of macrophage activation on their nanoparticle uptake. They demonstrated that activated macrophages (RAW 264.7 cell line) exhibited a significantly higher degree of nanoparticle uptake when compared to resting macrophages and that the therapy could decrease nitric oxide and pro-inflammatory cytokines production.

Zhao et al. went a step further and developed methotrexate-loaded (MTX) PLGA-PK3 (a novel polyketal) nanoparticles covered with folate-PEG, phosphatidylcholine and hydrophobic derivatives of octa-arginine with stearic acids (StaR8) [31]. Both folate and StaR8 synergistically promoted nanoparticle uptake by the activated macrophages (RAW 264.7 cell line) via endocytosis and membrane penetration mechanisms, respectively. In addition, they obtained a faster MTX liberation at pH 5.0 compared to pH 7.4 due to the properties of PLGA-PK3 core. Studies on an adjuvant-induced arthritis (AIA) rat model demonstrated that the administration of these nanoparticles almost fully restored the normal phenotype due to the MTX selective liberation inside the endosomes or lysosomes of activated macrophages, reducing pro-inflammatory cytokines production in vivo.

MTX is a disease modifying antirheumatic drug (DMARD) and is the first option for treating rheumatoid arthritis. For this reason, Kayat et al. developed MTX-loaded carbon nanotubes (CNTs) and functionalized them with folate because pristine CNTs cannot be used without functionalization [45]. They reduced the arthritic phenotype of carrageenan-induced arthritis male Albino rats in vivo and this effect was sustained for the 48 h of the study.

Moreover, MTX can also be used for prophylaxis, as shown by Nogueira et al. [44]. They encapsulated MTX in liposomes, which were composed of 1,2-dioleoyl-sn-glycero-3-phosphoethanolamine, cholesterol and *N*-(carbonyl methoxypolyethylene glycol-2000)-1,2-distearoyl-sn-glycero-3-phosphoethanolamine (DOPE/CH/DSPE-MPEG) and contained folic acid linked to a hydrophobic fragment of surfactant protein D. They demonstrated that the in vivo administration of that liposomal formulation in arthritic mice before the disease onset, prevented the development of symptoms while the administration of soluble MTX did not. This discovery may have an enormous impact on clinical practice because the prolonged use of MTX ultimately generates resistance, and therefore, this could be a way to delay or fully avoid it.

Overexpression of FR-β not only allows the active targeting of drugs, but also the development of cancer-analogue immunotherapies. FR-overexpressing cancer cells can be marked by highly immunogenic folate-conjugated haptens. Thus, if the animal is immunized against that hapten, it will generate an immune response against the whole cancer cell. In a previous review, Paulos et al. confirmed the effect of this immunotherapy against activated macrophages, which are located at inflamed regions, reducing the systemic symptoms of arthritis, regardless of the in vivo model tested [46]. These similitudes may allow hypothesizing about the results of Yang et al. when applied to this field: the group designed a thermosensitive polymeric folate-conjugated hydrogel (FA/PEA/DNA/PECE composite hydrogel) for the local delivery of tumor suppressor genes [27]. They demonstrated that the construct could release the desired genes in a controlled and sustained manner. These molecule-releasing hydrogels may have multiple applications in the regenerative medicine and tissue engineering fields.

In recent years, the use of small interfering RNAs (siRNAs) has gained popularity for their potential gene therapy applications. For the first time, Yang et al. achieved siRNA delivery against activated macrophages (RAW 264.7 cell line) by using folic acid-conjugated chitosan nanoparticles [25]. In vitro studies showed that nanoparticles were biocompatible, had preference against this cellular population, and the uptake was FR-β dependent. Anti-COX2 siRNA-loaded nanoparticles showed an 87% decrease in COX2 protein expression by the RNAi pathway, highlighting the importance of this therapeutic strategy. In the LPS-induced subcutaneous inflammation model used for in vivo testing, no nonspecific accumulation in inflamed tissues or tumors was observed, which could be due to the >100 nm diameter of the nanoparticles. For this reason, the authors proposed that their presence in inflamed areas may be the result of the co-migration with activated macrophages to these zones.

Apart from all these possible treatments for inflammatory diseases, some contrast agents have emerged for tracking the activated macrophages population by imaging techniques. Zhong et al. synthesized super paramagnetic iron oxide nanoparticles (SPIONs) with folic acid conjugated to PEG-*b*-PAA (polyamide amine), which allowed them to obtain magnetic resonance images (MRI) thanks to the magnetic properties of the nanoparticle core [28]. This system achieved good results with little secondary accumulation in the spleen and lungs, due to the mononuclear phagocyte system, and in a much lesser degree in the heart, kidney and liver.

In all the cases referred to above, folic acid was used as an active target agent to direct the effect of other molecules against activated macrophages. However, Samblas et al. recently published a study where they analyzed the effect of certain methyl donors, such as folic acid or vitamin B-12, in the THP-1-derived macrophages inflammatory response [26]. This is a human monocytic cell line derived from peripheral blood. However, for this reason, the results cannot be extrapolated to physiologic conditions. They showed that by pre-treating resting macrophages with these vitamins before adding lipopolysaccharide (LPS) to induce their activation, their inflammatory response decreased due to specific methylation of pro-inflammatory genes CpG sites. In addition, cytokine and chemokine expression also decreased, which led to a reduction in monocyte migration towards inflamed tissues.

Similarly, Weiss et al. explored the effects of exposing monocytes to nicotinamide (a vitamin B_3_ derivative), during their differentiation towards granulocyte macrophages (GM-MØ) in the presence of granulocyte macrophage colony-stimulating factor (GM-CSF), or macrophages (M-MØ) in the presence of macrophage colony-stimulating factor (M-CSF) [32]. They demonstrated that when vitamin B_3_ is present during the differentiation of monocytes to GM-MØ, NAM strongly interfered and reduced the pro-inflammatory features of GM-MØ, which included morphology and cytokine response changes as well as a decrease in the pro-inflammatory cytokines profile expression. Nevertheless, NAM did not influence M-MØ differentiation. Taken all together, it seems clear that more scientific research is needed in order to fully understand the molecular mechanisms involved in these processes.

### 3.3. B-Vitamins in Nervous System Repair

Regeneration of an injured nervous system is a complex objective due to the characteristics of the tissue. It is well-known that the peripheral nervous system has the capacity to physiologically regenerate damaged axons. On the other hand, it has been thought for decades that the central nervous system lacks this ability. However, nowadays it has been proved that there are certain areas of this system where active neurogenesis takes place, such as the sub ventricular zone of the lateral ventricle and the sub granular zone of the dentate gyrus in the hippocampus [51].

Folic acid plays a pivotal role during nervous system embryonic development, preventing problems in the neural tube closure or other developmental diseases [63]. However, its effect is not only limited to the embryonic period; it also has a role in tissue regeneration and repair processes in both the central and peripheral nervous system.

On the one hand, regarding the central nervous system, Iskandar et al. demonstrated that the intraperitoneal administration of folic acid increased axons repair up to 10-fold, including their functional recovery [50]. Hence, the team determined that the improvement was in part due to an epigenetic mechanism. These authors showed that after a spinal cord and peripheral nerve injury occur, folate receptor 1 (FolR1) increases its expression levels, which leads to a folate accumulation within cells [48]. Other groups have found that folic acid acts through methyl donors such as S-adenosylmethionine (SAM) and S-adenosylhomocysteine (SAH), regulating the DNMT enzymatic activity. This methyltransferase in turn activates tissue specific genes and the PI3K/Akt/CREB pathway in order to promote neural stem cell (NSC) proliferation. Furthermore, it has been shown that the increase in the DNMT activity due to folic acid administration potentiated NSC differentiation towards neurons and inhibited it towards astrocytes, while DNMT-inhibitor administration induced the opposite effect [12,13,14].

Taking into consideration all these findings, folic acid could be used as an adjuvant in cellular therapy that employs NSCs, as shown by Liu et al. This was the first study which reflected the effect of this vitamin on the proliferation of exogenous transplanted NSCs, in this case in a middle cerebral artery occlusion (MCAO) rat model [47]. In addition, 21 days after the administration of the therapy, they observed that exogenous cells had migrated to the infarcted region, suggesting another role in the migration of this cell population. However, folic acid by itself also constitutes a great defense against this disease. As investigated by Zhang et al., folic acid administration (28 days before and 14 days after MCAO) prevented cognitive function impairment (measured by Y-maze performance) after a cerebral ischemia event had occurred [51].

On the other hand, regarding the peripheral nervous system and emulating the experiments of Iskandar et al., Harma et al. assessed the effects of the intraperitoneal administration of folic acid on tibial nerve repair in rats [52]. A folate dose of 80 µg/kg gave the best results, significantly reducing latency values (which indicated axonal repair and improvement in nerve conduction), and enhancing the number and intensity of axons.

**Figure 4:**
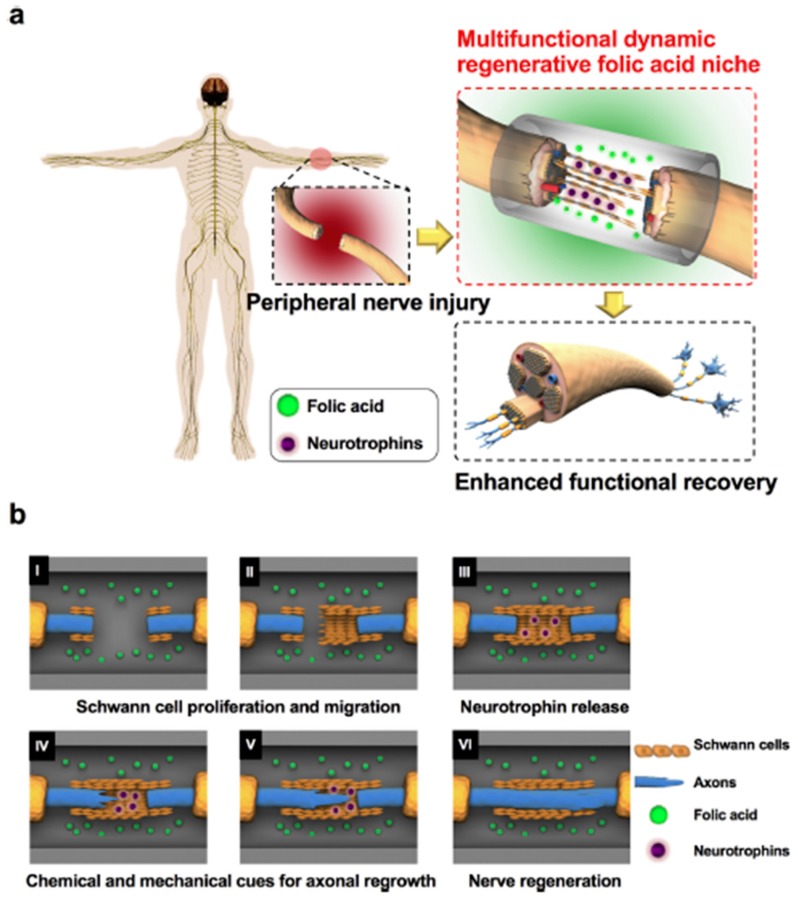
Nerve guidance conduits for the local release of folic acid in order to promote nerve regeneration. (**a**) Overview of the folic acid niche that provides chemical and mechanical cues to enhance functional recovery in a peripheral injury model. (**b**) Cartoon illustrating the detailed mechanism of the multifunctional and dynamic neuroregenerative folic acid niche and how it allows peripheral nerve regeneration by modulating the proliferation and migration of Schwann cells (**I**,**II**), stimulating the Schwann cells to release more neurotrophins (**III**), and increasing mechanical forces in the neurons that in turn boost the axonal regeneration of the neurons (**IV**,**V**) and peripheral nerve regeneration (**VI**). This figure is reproduced from [33] with permission from Elsevier Ltd.

More recently, Kim et al. developed nerve guidance conduits (NGCs), which were able to locally release folic acid [33]. This delivery system was made of biodegradable crosslinked urethane-doped polyester (CUPE) and it was designed to allow Schwann cell proliferation and migration, and the release of neurotrophins in order to promote axonal regeneration. They assessed their therapy in Wistar rats with >20 mm peripheral nerve injuries, and obtained promising results (Figure 4). CUPE NGCs allowed Schwann cells’ proliferation and migration as well as PC-12 migration and differentiation, due to methylation changes and chemical-to-mechanical force transduction. Moreover, they promoted neurotrophin-3 (NT-3) and NT-4/5 release, improving Schwann cell migration and peripheral nerve regeneration. The authors hoped to optimize the system to achieve a more controlled folic acid release because in this study they chose the dipping method to prepare folic acid-CUPE NGCs and it dissipated slowly during the 12-week experiment. Nevertheless, 8 weeks after the implantation, they obtained functional and nerve conduction results similar to those obtained by the autograft (positive control).

Finally, an important consequence of nerve injuries is the associated neuropathic pain (NeP). It has been demonstrated that folic acid and vitamin B_12_ play an important role in decreasing NeP in peripherical neuropathy [64]. Many molecular mechanisms involved in this pain are still unknown. Nevertheless, it is known that after a spinal cord injury (SCI) occurs, matrix metalloproteinase-2 (MMP2) expression induces NeP. In addition, many other MMPs are implicated in the process of matrix remodeling and glial scar formation. For this reason, and taking into account the abovementioned epigenetic role of folic acid in tissue specific genes, Miranpuri et al. assessed the effect of intraperitoneal administration of folic acid in Sprague-Dawley rats with SCI [49]. They demonstrated that folic acid alleviated neuropathic pain symptoms and it diminished MMP2 expression levels and increased functional recovery after SCI. However, although they proposed an epigenetic mechanism, they also raised the need for more in-depth study of the molecular mechanisms that alter MMP2 expression levels.

Apart from folic acid, other B-vitamins have also demonstrated their excellent properties for achieving successful nervous system repair. In this regard, Griffin et al. studied the effect of nicotinamide, an active form of vitamin B_3_, when applied in vitro to mouse embryonic stem cells (mESCs) [34]. After a 14-day exposure, they concluded that certain doses of NAM promoted mESCs differentiation towards a mature neuronal phenotype; revealing the potential of vitamin B_3_ as an additional tool in nerve regeneration therapies.

Nevertheless, in a study conducted by Al-saaeed et al., where they tested the regenerative potential of vitamins B_1_, B_6_ and B_12_ on a sciatic nerve injury model in vivo, it was demonstrated that cobalamin (vitamin B_12_) had the most effective regenerative activity amongst all assayed B-vitamins [53].

For this reason, Suzuki et al. evaluated whether local administration of methylcobalamin (MeCbl) at the nerve injury was effective for promoting nerve regeneration [36]. They synthesized MeCbl-loaded nanofibers by electrospinning, which gradually released the vitamin for up to 8 weeks and implanted them in a rat model of sciatic nerve injury. This showed no adverse effects and had no effect on MeCbl plasma concentration but it accelerated the functional recovery, measured in terms of nerve conduction velocity and myelination.

In addition, taking advantage of the vitamin B_12_ properties in nerve regeneration, Romano et al. investigated the effects of an ophthalmic solution containing vitamin B_12_ in rat models with an induced corneal injury [54]. The solution was composed of vitamin B_12_ 0.05% plus taurine 0.5% and sodium hyaluronate 0.5% and was applied to the rats’ right eye; whilst the left eye was used as the control being treated with a solution containing taurine 0.5% and sodium hyaluronate 0.5%. In both cases, the solution was applied 4 times a day for 10–30 days. The results displayed that local treatment with a solution containing vitamin B_12_ led to faster repair of corneal damage, and that vitamin B_12_ is particularly effective in promoting mechanisms that facilitate reinnervation.

Furthermore, eye damage can also appear as a result of type-II diabetes. Diabetic retinopathy (DR) is already one of the most common causes of vision loss among people with diabetes and a leading cause of blindness among young adults. Scutellarin (Scu) has demonstrated its effectiveness in treating DR but its low bioavailability has limited its clinical use. For this reason, Wang et al. developed vitamin B_12_-modified amphiphilic chitosan derivative (Chit-DC-VB_12_) nanoparticles loaded with Scu in order to improve its bioavailability and to achieve a more effective treatment [35]. In vivo results obtained by the group showed that both objectives were accomplished, down-regulating the central retinal artery resistivity index and angiogenic proteins expression such as VEGF, VEGFR2 and vWF on type II-diabetic rats’ retinas.

### 3.4. Vitamin B in Other Tissue Repair

Apart from the applications outlined above, there are other tissues where B-vitamin derivatives have potential use as regenerative molecules.

#### 3.4.1. Dermal Repair

Wound healing in injured skin tissue. consists of the inflammation, proliferation and remodeling stages. Although many factors have been demonstrated to promote this physiological process, there are some cases where the regenerative process is especially difficult, e.g., in diabetic ulcers [65]. For this reason, Xiao et al. synthesized copper metal-organic framework nanoparticles which were stabilized with folic acid (FA-modified HKUST-1) and that released Cu^2+^ for its pro-angiogenic potential [37]. They determined that folic acid slowed Cu^2+^ liberation down, reducing toxicity and increasing in vitro keratinocytes and fibroblasts migration. Moreover, using an in vivo model, they achieved an increase in angiogenesis, collagen deposition, re-epithelization and in the wound healing rate.

Apart from its regulatory role, folinic acid, which is one of the natural forms of folate in food, has been demonstrated to accelerate the wound healing rate in rats [55]. In particular, Duman et al. concluded that 2.5% topical folinic acid was the optimum concentration for achieving this effect, which comprises an increase in skin firmness and collagen, pro-collagen, MMP-1 and MMP-9 expression levels. In addition, the number of mast cells which migrate towards the injured area is also augmented, stimulating fibroblasts to promote wound healing and affecting collagen maturation and the remodeling phase.

Alongside folic acid, pantothenic acid (B_5_ vitamin) is known for supporting fibroblast migration and proliferation, and consequently, for promoting wound healing [66]. Accordingly, Fan et al. focused on the usefulness of fabricating silk-fibroin nanofibrous matrices reinforced by VB_5_ and explored its biomedical applications [38]. These composite nanofibrous matrices were successfully manufactured and they increased cell viability in the L929 fibroblast cell line in long-culture periods, and specifically, assisted skin cell survival under oxidative stress conditions; revealing the potential properties of pantothenic acid for skin care products.

Lastly, Rodgers et al. have demonstrated that hydrogels have excellent properties for use in tissue engineering since they mimic the conditions of host tissues. Although a non-invasive injection is preferred, this requires the solidification of the gel in vivo when exposed to endogenous stimuli, which is sometimes troublesome. Nevertheless, there has been recent developments in light-responsive agents for targeted therapies using vitamin B_12_ as the ultimate component for regenerative therapies [39] and Rodgers et al. took advantage of these modern photopolymerizing techniques in their research [39]. However, this technique faces some issues when used for dermal repair, because the biological chromophores of the inner body compete for light with the endogenous chromophores; meaning the curing effect is limited to a few millimeters into the skin, although the healing potential is increased as there is more melanin there. Rodgers et al. demonstrated that light-induced hydrogel formation can be mediated by alkyl-cobalamin-based (vitamin B_12_) photo-initiators extending the wave-length from green to red, thus, resolving the differences in curing depth between low and high melanin skin.

Wang et al. developed a completely recombinant protein-based hydrogel by covalently assembling adenosylcobalamin-dependent photoreceptor (AdoB_12_) and C-terminal adenosylcobalamin binding domain (CarH_C_) proteins, which undergo gel-sol transition when exposed to light due to the disassembly of CarH_C_ [40]. This was used as a carrier for cells such as fibroblasts and MSCs. Results showed that within 5 min, gels had completely undergone the transition alongside a complete release of the cells, and that the majority of the cells remained viable; highlighting the potential use of these hydrogels as protein and cell carriers.

#### 3.4.2. Pancreatic Repair

Diabetes mellitus is a group of metabolic diseases characterized by defects in insulin secretion, action or both. Due to the rise in prevalence over the last three decades, diabetes is now considered to be the epidemic of the century [67]. In this framework, Ilie et al. developed a new method for enhancing insulin production based on multi-walled carbon nanotubes (MWCNTs) functionalized with nicotinamide (NAM-MWCNTs), which acted via the macrophage migration inhibitory factor (MIF) pathway [41]. This factor and insulin colocalize within the secretory granules of the pancreatic islet beta-cells, and once they are released, also appear to regulate the insulin release in an autocrine fashion. Several quantification methods revealed that cultures treated with NAM-MWCNTs had an almost 90% increase in insulin production when compared to NAM-treated cells, which increased production by only 50%; revealing a strong interaction between NAM-MWCNTs and the MIF signaling pathway, and regulating insulin secretion in β-pancreatic cells. For this reason, NAM-MWCNTs may be tested in vivo to determine if it would be useful as a pancreatic regenerative therapy because it could restore the normal function of the organ.

#### 3.4.3. Cardiovascular Repair

It is well-known that nitric oxide (NO) accelerates cardiac regeneration by modulating the inflammatory response [68]. Thus, developing NO-delivery systems may be an interesting option for regenerative purposes. In this context, Wan et al. synthesized NO-releasing chitosan-folate conjugates [69]. Briefly, carboxylic groups of folic acid reacted with the NH_2_ groups of chitosan and then, the secondary amine reacted with high pressures of NO (5 atmospheres) for generating the N-diazeniumdiolates of NO donors, which have been demonstrated to spontaneously release NO under physiological conditions. Apart from cardiac regeneration, this macromolecular complex could be used in many situations where NO plays a positive role (for example, cancer, wound healing, etc.).

Recently, Li et al. used folic acid as an adjuvant in a cell therapy based on induced pluripotent stem cells (iPSCs) for cardiac regeneration after an acute myocardial infarction [42]. After modifying folic acid with a specific peptide sequence (FA-FFFRGD-ss-EE, FA-Seq), they used it as a pro-gelator to synthesize the injectable hydrogel. They determined that 1% wt FA-Seq not only did not inhibit iPSCs proliferation, but also promoted their differentiation towards cardiac lineage. In vivo studies on infarcted C57BL/6 mice demonstrated the feasibility of the hydrogel for enhancing the effect of the cellular therapy in a localized manner, for minimizing associated fibrosis and for improving cell survival, neovascularization and cardiac activity after 30 days.

Angina pectoris is a medical condition that occurs when the heart takes a lower volume of oxygenated blood. It is normally treated with nicorandil but this has limitations, including its low bioavailability and the mucosal ulcerations that it generates. Not only can vitamin B be used as a tool for cardiovascular repair, but also as a carrier for anti-anginal drugs to increase its bioavailability. This is why Singh et al. developed a novel biocomposite polymeric nanofiber composed of vitamin B_12_, hyaluronic acid and polyvinyl alcohol (PVA), which was then loaded with the drug for sublingual delivery [56]. The pharmacokinetics of the sublingual administration were compared to the marketed oral formulation, and showed comparable therapeutic efficacies while avoiding the mucosal ulcerations.

## 4. Materials and Methods

An extensive search was executed in PubMed (NCBI U.S. National Library of Medicine), Science Direct^©^ (Elsevier) Web of Science™ and SCOPUS. The search procedure was carried out using a combination of different keywords, such as “Vitamin B”, “Folic acid”, “Tissue engineering”, “Regenerative medicine”, “Scaffold”, “Stem cells”, “Differentiation”, “Nanoparticle”, “Regeneration”, “Repair”, and analogous and complementary terms that might be useful to the study. The majority of the results were focused on cancer and vitamin B nutritional supplements, and therefore, they were almost entirely discarded as they were considered beyond the scope of this review. Only articles that were focused on tissue regeneration strategies, and which were aimed at structural and/or functional restoration were reviewed. These articles were classified according to the target tissue or pathology and divided into in vitro and/or in vivo studies as shown in Figure 1. 

## 5. Expert’s Opinion on Future Directions and Conclusions

In this article, the latest advances in functional mechanisms and current applications in regenerative medicine based on folic acid and other vitamin B derivatives have been reviewed. To the best of our knowledge, this is the first systematic review on this topic and all the investigations cited above allowed us to conclude that B-vitamins are excellent adjuvants to achieve regeneration in several types of tissue. Not only have B-vitamins been used to improve the mechanical properties of existing biomaterials (hydrophilicity, biocompatibility, etc.), but also to actively target different drugs for the treatment or prophylaxis of some diseases, and to exert their own effects on tissues, e.g., to increase proliferation or induce differentiation processes.

From the authors’ point of view, folic acid derivatives are considered one of the most encouraging derivatives because they fulfill the requirements for their application in clinical use. First of all, these derivatives are highly stable under standard conditions and they do not have special storage requirements. In addition, they have been shown to promote regeneration processes in a wide range of tissues and organs with a long lifespan in physiological conditions, which is the greatest limitation of other regeneration therapies such as the use of growth factors or other recombinant proteins. In addition, access to folic acid from natural resources permits the preparation of these derivatives at relatively low prices. Finally, as folic acid and other B-vitamins are well-known, safe compounds, they are less regulated compared to other drugs trying to reach the market. These factors could easily capture pharmaceutical interests.

## Figures and Tables

**Table 1:** In vitro studies using B-vitamins-based regenerative therapies.

Target Tissue	Compound	Method	Specie	Cell Line	Results	Ref.
Musculoskeletal regeneration	SrFO	SrFO-functionalized Ti substrates	Murine	Primary MSCs	Promoted cell adhesion, proliferation and osteogenic differentiation of MSCs	[15]
		SrFO-loaded biohybrid scaffolds	Human	Dental pulp human MSCs	Enhanced osteogenic related gene expression and osteogenic differentiation	[16]
	SrFO and CaFO	Sr- and Ca-based folates	Human	HOBs	Overexpression of ALP activity	[17]
	Folic acid	Hydroxyapatite nanoparticles loaded with FA	Human	Human MSCs	Overexpression of ALP activity and *Runx2* gene (osteogenic differentiation)	[18]
		Vancomycin release from folic acid/ZnO quantum dots sealed titania nanotubes	Bacteria	*Staphylococcus aureus*	Prevented Ti implants-associated bacterial infections	[19]
	PABA	PABA doped micro-grooved carbon fibers	Murine	L929 and MC3T3-E1	Promoted the adhesion and proliferation of pre-osteoblasts with minimized cytotoxicity	[20]
	Riboflavin and derivatives	Free riboflavin and its derivatives	Murine	MC3T3-E1	Promoted osteoblastic differentiation, enhancing the effects of the typical inductors	[21]
	Nicotinamide	Free nicotinamide	Murine	Primary rat bone marrow cells	Protected against oxidative-stress-related cytotoxicity	[22]
	Vitamin B_6_	Pyridoxine and pyridoxal forms on PLA nanofibers	Monkey Canine Human	Cos-7, MDCK, and HEp-2 cells	Protected against oxidative stress	[23]
		Pyridoxal 5′-phosphate-immobilized TiO_2_ surfaces	Murine	MC3T3-E1	Improved cell proliferation, platelet aggregation and activation and blood coagulation	[24]
Inflammatory diseases	Folic acid or Folate	FA-conjugated chitosan for siRNA delivery	Murine	RAW 264.7	Enhanced cellular uptake and silencing effect of siRNAs	[25]
		Free folic acid	Human	THP-1	Folic acid pre-treatment diminished the inflammatory response in LPS-activated THP-1 macrophages	[26]
		FA/PEA/DNA/PECE composite hydrogel	Murine	C26 and 293T	Potential vector for gene delivery	[27]
		FA conjugated PEG-*b*-PAA@SPION	Murine	RAW 264.7	Promoted cellular uptake in activated macrophages	[28]
		Folate-targeted dendrimers	Murine	RAW 264.7	Promoted FR-mediated cellular uptake in activated macrophages	[29]
		Dexamethasone phosphate-loaded folate-conjugated polymeric nanoparticles	Murine	RAW 264.7	Promoted cellular uptake in activated macrophages and inhibits the production of pro-inflammatory cytokines and NO	[30]
		pH-responsive nanoparticles decorated with cell penetrating peptide and folate	Murine	RAW 264.7	Promoted FR-mediated cellular uptake in activated macrophages	[31]
	Nicotinamide	Free nicotinamide	Human	Primary monocytes	Reduced pro-inflammatory features of GM-MØ	[32]
Nervous System Repair	Folic acid	FA-loaded-CUPE nerve guidance conduits	Murine	Rat Schwann and PC-12 Adh cells	FA induced NT-3, NT-4/5 release and promoted proliferation and migration of both cell lines and differentiation of PC-12	[33]
		Free folic acid	Murine	Neonatal Sprague-Dawley NSCs	Promoted NSCs proliferation by a DNMT-and dose-dependent mechanism	[12]
			Murine	Neonatal Sprague-Dawley NSCs	Promoted NSCs proliferation by epigenetic regulation of PI3K/Akt/CREB pathway	[13]
			Murine	Neonatal Sprague-Dawley NSCs	Promoted neural and decreases astrocytic differentiation in NSCs by regulating DNMT	[14]
	Nicotinamide	Free nicotinamide	Murine	mESCs	Induced neuronal differentiation	[34]
	Cyanocobalamin (Vitamin B_12_)	B_12_-modified amphiphilic chitosan nanoparticles loaded with scutellarin	Human and zebra fish	Caco-2 cells and zebra fish	Showed good biocompatibility and high permeation in human cells	[35]
	Methylcobalamin (MeCbl)	MeCbl-loaded nanofibers	Murine	Primary cortical neurons	MeCbl promoted axonal outgrowth and the nanofibers released it gradually for up to 8 weeks	[36]
Dermal Repair	Folic acid	FA-modified HKUST-1	Human	HEKs, HDFs and HUVECs	FA reduced cytotoxicity while enhancing cell migration	[37]
	Pantothenic acid	Pantothenic acid/silk fibroin composite nanofibers	Murine	L929 cells	Especially promoted skin cells survival under oxidative stress conditions	[38]
	Alkyl-cobalamin (Vitamin B_12_)	B_12_-mediated photo-polymerized hydrogels	Human	HepG_2_ cells	Allowed cell survival	[39]
	Cyanocobalamin	B_12_-dependent photoresponsive protein hydrogels	Human	3T3 fibroblasts and human MSCs	Allowed facile release/recovery of the cells from 3D cultures without comprising their viability	[40]
Pancreatic Repair	Nicotinamide	Multiwalled carbon nanotubes functionalized with nicotinamide	Human	Hybrid beta-cell line (1.4E7)	Enhanced insulin production via MIF pathway	[41]
Cardiovascular System Repair	Folic acid	FA-derived hydrogel	Murine	Mouse iPSCs	1% wt hydrogels promoted cell proliferation and did not affect iPSCs differentiation towards cardiac lineage	[42]

**Table 2:** In vivo studies using B-vitamins-based regenerative therapies.

Target Tissue	Compound	Method	Specie	Body Part	Results	Ref.
Bone Repair	SrFO	SrFO-functionalized Ti substrates	Sprague-Dawley Rat	Femur	Improved bone formation, especially in the later stages.	[15]
		SrFO-loaded biohybrid scaffolds	Wistar Rats	Skull	Increased new bone formation	[16]
	Vitamin B_6_	Pyridoxal 5′-Phosphate-immobilized TiO_2_ surfaces	Rat	Femur	Improved the hemophilicity for promoting osteointegration	[24]
Inflammatory diseases	Folic acid	MTX-loaded folate-tagged liposomes	DBA/1J mice	Joints	Prophylactic effect before the disease onset	[43,44]
		FA-appended surface-engineered multi-walled carbon nanotubes	Albino rats	Joints	Enhanced biodistribution and sustained MTX release	[45]
		FA-conjugated chitosan for siRNA delivery	Balb/c mice	Subcutaneous inflammation	Enhanced biodistribution and siRNA delivery to inflammation sites	[25]
		FA/PEA/DNA/PECE composite hydrogel	Tumor-bearing mice	Tumor	Potential vector for sustained gene release	[27]
		FA conjugated PEGb- PAA@SPION	Lewis rats	Joints	Potential candidate for the MRI of rheumatoid arthritis	[28]
		Folate-hapten conjugates	Different Models	Joints	Successful folate-targeted immunotherapy	[46]
		Folate-Targeted Dendrimers	C57BL6 mice and ApoE^(−/−)^ knockout mice	Inflammation sites	Enhanced biodistribution, being a potential drug carrier	[29]
		pH-responsive nanoparticles decorated with cell penetrating peptide and folate	Arthritis induced-Sprague-Dawley rats	Joints	Inhibited pro-inflammatory cytokines secretion and ameliorated systemic symptoms	[31]
Nervous System Repair	Folic acid	FA-loaded-CUPE nerve guidance conduits	Wistar rats	Right sciatic nerve injury (22 mm)	Improved nerve regeneration at 8 weeks	[33]
		Free folic acid + NSCs therapy	Sprague-Dawley rats	Brain (MCAO procedure)	FA stimulated transplanted NSCs proliferation and migration to ischemic zones	[47]
		Free folic acid or folate	Sprague-Dawley rats and *Folr1*^+/+^ or *Folr1*^+/–^ mice	Spinal cord	Spinal cord and peripheral nerve injuries increased *FolR1* expression. FA exerted its action by epigenetic mechanisms	[48]
			Sprague-Dawley rats	Spinal cord	FA alleviated NeP and improved functional recovery post-SCI, possibly by reducing the expression of MMP2	[49]
			Sprague-Dawley rats	Spinal cord	Improved the regrowth of sensory spinal axons	[50]
			Sprague-Dawley rats	Brain (MCAO procedure)	Enhanced notch signaling and hippocampal neurogenesis and diminished the impairment of cognitive function after a stroke	[51]
			Wistar albino rats	Tibial nerves	Improved peripheral nerve healing, with increased myelination and reduced fibrosis	[52]
	Vitamins B_1_, B_6_ and B_12_	Free vitamins B_1_, B_6_ and B_12_	Albino rats	Sciatic nerve	All B-vitamins promoted regeneration to some extent (B_12_ > B_1_ > B_6_)	[53]
	Cyanocobalamin	Ophthalmic solution: B_12_ 0.5% + sodium hyaluronate 0.5%	Wistar rats	Cornea	Accelerated the nerve repair and reinnervation processes	[54]
	Cyanocobalamin	B_12_-modified amphiphilic chitosan nanoparticles loaded with scutellarin	Sprague-Dawley rats	Retinas and retinal arteries	Improved scutellarin bioavailability, increasing the effects of the drug	[35]
	MeCbl	MeCbl-loaded nanofibers	Wistar rats	Sciatic nerve	Promoted functional recovery (nerve conduction velocity and myelination)	[36]
DermalRepair	Folinic acid	Folinic acid cream	Sprague-Dawley rats	Skin wounds	Improved wound healing and enhanced collagen synthesis and MMP1 and MMP9 expression	[55]
	Folic acid	FA-modified HKUST-1	Diabetic and non-diabetic mice (db/db and C57BL/6)	Skin wounds	Induced angiogenesis, promoted collagen deposition and re-epithelialization, and increased wound closure rates	[37]
	Alkyl-cobalamin	B_12_-mediated photo-polymerized hydrogels	Dermal Tissue Model	Skin	This synthesis method extended the curing wavelength from green to red, resolving the healing differences between low and high melanin skins	[39]
Cardio-vascular System Repair	Folic acid	FA-derived hydrogel	C57BL/6 mice	Myocardium	Improved neovascularization and cardiac function as well as eased post-myocardial-infarction-associated fibrosis	[42]
	Cyanocobalamin	Nicorandil-loaded nanofibers composed of vitamin B_12_, hyaluronic acid and PVA	Wistar rats	Local tissue (sublingual administration)	The composite avoided the adverse effects of the oral administration (mucosal ulceration) with an effectiveness comparable to the commercialized one	[56]

## Abbreviations

AdoB_12_	Adenosylcobalamin
AIA	Adjuvant-induced arthritis
ALP	Alkaline phosphatase
CaFO	Calcium folate
CarH-C	C-terminal AdoB_12_ binding domain
CF	Carbon fibers
Chit-DC-VB_12_	B_12_-modified amphiphilic chitosan derivative
CNT	Carbon nanotubes
CREB	cAMP Response Element Binding
CUPE	Crosslinked urethane-doped polyester
DMARD	Disease modifying antirheumatic drug
DNMT	DNA methyltransferase
DR	Diabetic retinopathy
FA	Folic acid
FA-Seq	Folic acid-FFFRGD-ss-EE (peptide sequence)
FolR1	Folate receptor-1 isoform
FR-β	Folate receptor-β isoform
GM-CSF	Granulocyte macrophage colony-stimulating factor
GM-MØ	Granulocyte macrophages
HA	Hydroxyapatite
HOBs	Human osteoblasts
HKUST-1	Copper metal-organic framework nanoparticles
iPSCs	Induced pluripotent stem cells
LPS	Lipopolysaccharide
M-CSF	Macrophage colony-stimulating factor
M-MØ	Macrophages
MCAO	Middle cerebral artery occlusion
MeCbl	Methylcobalamin
mESCs	Mouse embryonic stem cells
MIF	Macrophage migration inhibitory factor
MMPs	Matrix metalloproteinases
MRI	Magnetic resonance imaging
MSCs	Mesenchymal stem cells
MTX	Methotrexate
MWCNTs	Multiwalled carbon nanotubes
NAM	Nicotinamide
NeP	Neuropathic pain
NGCs	Nerve guidance conduits
NO	Nitric oxide
NPs	Nanoparticles
NSCs	Neural stem cells
NTs	Nanotubes
PAA	Polyamide amine
PABA	Para-amino benzoic acid
PAMAM	Polyamidoamine
PEA	Poly (ester amine)
PECE	PEG_550_-PCL_2200_-PEG_550_
PEG	Polyethylene glycol
PIP3K	Phosphatidylinositol-4,5-bisphosphate 3-kinase
PLA	Polylactic acid
PLGA	Poly (lactic-co-glycolic acid)
PLP	Pyridoxal 5′-phosphate
PVA	Polyvinyl alcohol
QD	Quantum dots
ROS	Reactive oxygen species
SAH	S-adenosylhomocysteine
SAM	S-adenosylmethionine
SCI	Spinal cord injury
Scu	Scutellarin
siRNA	Small interfering ribonucleic acid
SPIONs	Super paramagnetic iron oxide nanoparticles
SrFO	Strontium folate
TCP	Tricalcium phosphate
TNT	Titania nanotubes
VEGF	Vascular endothelial growth factor
VEGFR2	Vascular endothelial growth factor receptor 2
vWF	von Willebrand factor
